# Analysis of Conjunctival Sac Microbiome in Dry Eye Patients With and Without Sjögren's Syndrome

**DOI:** 10.3389/fmed.2022.841112

**Published:** 2022-03-08

**Authors:** Hang Song, Kang Xiao, Zhengyu Chen, Qin Long

**Affiliations:** ^1^Department of Ophthalmology, Peking Union Medical College Hospital, Beijing, China; ^2^Key Laboratory of Ocular Fundus Diseases, Chinese Academy of Medical Sciences & Peking Union Medical College, Beijing, China

**Keywords:** ocular surface, microbial diversity, microbial composition, dry eyes, Sjögren's syndrome

## Abstract

**Purpose:**

To analyze the conjunctival sac microbial communities in patients with Sjögren's syndrome-associated dry eyes (SSDE) and non-Sjögren's syndrome-associated dry eyes (NSSDE), compared with normal controls (NC).

**Methods:**

Conjunctival sac swab samples from 23 eyes of SSDE, 36 eyes of NSSDE, and 39 eyes of NC were collected. The V3–V4 region of the 16S ribosomal RNA (rRNA) gene high-throughput sequencing was performed on an Illumina MiSeq platform and analyzed using Quantitative Insights Into Microbial Ecology (QIIME). Alpha diversity was employed to analyze microbiome diversity through Chao1 and Shannon indexes. Beta diversity was demonstrated by the principal coordinates analysis (PCoA) and Partial Least Squares Discrimination Analysis (PLS-DA). The relative abundance was bioinformatically analyzed at the phylum and genus levels.

**Results:**

The alpha diversity was lower in patients with dry eye disease (Shannon index: NC vs. SSDE: *P* = 0.020, NC vs. NSSDE: *P* = 0.029). The beta diversity showed divergent microbiome composition in different groups (NC vs. SSDE: *P* = 0.001, NC vs. NSSDE: *P* = 0.001, NSSDE vs. SSDE: *P* = 0.005). The top 5 abundant phyla were Firmicutes, Proteobacteria, Actinobacteriota, Bacteroidota, and Cyanobacteria in all three groups. The top five abundant genera included *Acinetobacter, Staphylococcus, Bacillus, Corynebacterium*, and *Clostridium_sensu_stricto_1*. The relative microbiome abundance was different between groups. The Firmicutes/Bacteroidetes (F/B) ratio was 6.42, 7.31, and 9.71 in the NC, NSSDE, and SSDE groups, respectively (NC vs. SSDE: *P* = *0.0*38, NC vs. NSSDE: *P* = *0.9*91, SSDE vs. NSSDE: *P* = 0.048).

**Conclusion:**

The diversity of conjunctival sac microbiome in patients with NSSDE and SSDE was diminished compared with NC. The main microbiome at the phylum and genus level were similar between groups, but the relative abundance had variations. The Firmicutes/Bacteroidetes ratio was higher in the SSDE group.

## Introduction

Dry eye disease (DED) is a multifactorial inflammatory ocular disease featured by tear film instability and hyperosmolarity, inflammation, and neurosensory abnormalities (1, 2). Symptoms include burning, foreign body sensation, photophobia, and blurred vision, which would adversely affect daily lives (3). Autoimmune disease-associated dry eye is one of the most important types of DED (4). Sjögren's syndrome (SS) is a chronic autoimmune disease featured by dry mucosal surfaces and other systemic muscular pain, and dry eye is one of the most discomforting symptoms that patients with SS complain about (5, 6). The lacrimal gland becomes infiltrated with activated CD4+ T cells and B cells, and ocular surface diseases develop from reduced lubrication, as well as from cytokines produced by activated epithelial cells and infiltrating inflammatory cells (7, 8).

Recently, mounting evidence shows that microbial imbalance changes the immunity toward an increased inflammatory response, which is closely associated with the occurrence of multiple chronic inflammatory diseases (9, 10). Hence, it is hypothesized that similar mechanisms are also active on the ocular surface and are involved in the pathophysiology of DED including SS. The production of lipases and toxins by different proportions of colonizing bacteria may destabilize the lipid layer of the tear film, interact with the conjunctival mucins (11, 12) and cause ocular surface cellular damage. Thus, dysbiosis might be associated with tear film instability, inflammation, and ocular irritation.

The purpose of the study was to explore the potential changes in the conjunctival sac microbiome in dry eye patients with and without SS, compared with healthy subjects. This may help to better understand its pathophysiology and provide more evidence to develop new treatment strategies.

## Methods

### Sample Collection

Patients were recruited from the regular outpatient clinic, Department of Ophthalmology, Peking Union Medical College Hospital, between September 1 and October 30, 2021. Patients with SS-associated dry eyes (SSDE), non-SS-associated dry eyes (NSSDE), and normal controls (NC) with healthy ocular surface conditions were recruited. Dry eye was diagnosed based on the Chinese Expert Consensus on dry eyes (13) as follows: patients presenting with dry eye symptoms, such as burning, foreign body sensation, blurred vision, and photophobia; Ocular surface disease index (OSDI) ≥13; Fluorescein breakup time (FBUT) ≤ 5 s or Schirmer's test (without local anesthesia) ≤ 5 mm/5 min. If 5 s <FBUT test ≤ 10 s and 5 mm/5 min <Schirmer's test (without local anesthesia) ≤ 10 mm/5 min, patients with cornea staining score ≥5 were also diagnosed with dry eye. The cornea staining was scored by dividing the corneal surface into five areas as proposed by the US National Eye Institute (14), the punctate staining in each area was recorded as a score of 0–3, with 0: no staining; 1: <15 dots; 2: 16–20 dots; 3: >30 dots, strip/bulk staining or corneal filaments. The final score is the sum of the scores from the five areas. All patients with SS had a complete ocular, oral, and rheumatologic evaluation, including the panel of serum autoantibodies, and met the SS classification criteria made by the American College of Rheumatology/European League Against Rheumatism (ACR-EULAR) (15). Patients with healthy ocular surface conditions were included as the NC group after ruling out dry eyes on a slit-lamp examination. The exclusion criteria included a history of uveitis, glaucoma, retinal disease, ocular trauma/transplantation in the previous 4 weeks, application of antibiotic or immunomodulatory eyedrops in the previous 4 weeks, and eye surgeries within 3 months. A sterile cotton swab was used to collect specimens by rubbing the swab from the medial to the lateral side of the inferior fornix of the conjunctival sac of each right eye without anesthesia. The swabs were then placed in sterile tubes and stored in a refrigerator (at −20°C) before further experiments. The study adhered to the tenets of the Declaration of Helsinki and was approved by the Institutional Review Board of Peking Union Medical College Hospital (ZS-3092). Informed consent forms were obtained from all patients.

### DNA Extraction, PCR Amplification, and 16S rRNA Gene Amplicon Sequencing

Deoxyribonucleic acid (DNA) was extracted using the MicroElute Genomic DNA Kit (D3096, Omega, MA, USA) according to the manufacturer's instructions. The concentration of DNA was measured using a NanoDrop 2000 ultramicro-spectrophotometer (Thermo Scientific, Waltham, MA, USA). The V3–V4 region of the 16S ribosomal RNA (rRNA) gene was amplified from tue extracted genomic DNA samples with primers (319 F: 5′-ACTCCTACGGGAGGCAGCAG-3′ and 806 R: 5′-GGACTACHVGGGTWTCTAAT-3′) manufactured by Sangon Biotech (Shanghai) Co., Ltd. PCR was carried out on a Mastercycler Gradient (Eppendorf, Germany) using 25 μl reaction volumes, containing 12.5 μl 2× Taq PCR MasterMixII (Vazyme Biotech Co., Ltd, China), 3 μl BSA (2 ng/μl), 1 μl Forward Primer (5 μM), 1 μl Reverse Primer (5 μM), 2 μl template DNA, and 5.5 μl ddH2O. The PCR amplification products were purified with Agencourt AMPure XP magnetic beads (Fisher Scientific, Hampton, NH, USA), dissolved in an elution buffer, and then labeled. The fragment range and the concentration of the library were detected using the Agilent 2100 Bioanalyzer (Agilent, Santa Clara, CA, USA). Qualified libraries were selected for sequencing on the MiSeq PE300 platform based on the size of the inserted fragments.

### Bioinformatic Analysis

Samples were sequenced on an Illumina MiSeq platform (Illumina, Inc., San Diego, CA, USA) according to the manufacturer's instructions. Qualified paired-end reads were separated using the sample-specific barcode sequences and trimmed with the Illumina Analysis Pipeline Version 2.6 (Illumina, Inc., San Diego, CA, USA). Then, the dataset was analyzed using the Quantitative Insights Into Microbial Ecology (QIIME) (Version 1.8.0; Boulder, USA). The sequences were clustered into operational taxonomic units (OTUs) at a similarity ≥97%, to generate rarefaction curves and to calculate the richness and diversity indices. The Ribosomal Database Project (RDP) Classifier tool was used to classify all sequences into different taxonomic groups.

Alpha diversity was employed to analyze the complexity of species diversity for each sample through Chao1, Shannon indexes generated by QIIME. Beta diversity was demonstrated by the principal coordinates analysis (PCoA) and Partial Least Squares Discrimination Analysis (PLS-DA) to evaluate the microbiome complexity between samples. The taxonomy and relative abundance were bioinformatically analyzed at phylum and genus levels.

### Statistical Analysis

The R software (Version 3.2.5; New Zealand) and GraphPad Prism 5.0 (GraphPad Software, San Diego, CA, USA) were used for the statistical analyses. Tukey test was used to identify significant between-group differences for alpha-diversity. The divergence between the two groups was compared by Analysis of Similarity (ANOSIM). The relative abundance of bacteria was compared by one-way ANOVA. The demographic and clinical data are expressed as mean ± SD. A *P*-value <0.05 was considered statistically significant.

## Results

### Demographic Characteristics of Patients

Samples were collected from 23 eyes of SSDE, 36 eyes of NSSDE, and 39 eyes of NC. The mean age was 48.09, 39.89, and 35.61 years old in the SSDE, NSSDE, and NC groups, respectively (Table 1). The average length of Schirmer's test was 5.72 ± 6.25 (mm/5 min) in the SSDE group and 6.68 ± 8.63 (mm/5 min) in the NSSDE group. The average FBUT was 2.32 ± 1.34 (s) in the SSDE group and 4.71 ± 2.79 (s) in the NSSDE group. The average cornea staining score was 6.67 ± 3.32 in the SSDE group and 2.32 ± 1.03 in the NSSDE group (Table 1).

**Table 1:** Demographic characteristics and basic clinical parameters.

		**SSDE**	**NSSDE**	**NC**	***P*-value**
Sex	Male/female	0/23	9/27	12/27	*P* = 0.001
Age	Mean ± SD (years)	48.09 ± 9.01	39.89 ± 13.45	35.61 ± 11.03	*P* = 0.008 SSDE vs. NSSDE, *P* = 0.007 NC vs. NSSDE, *P* <0.001, NC vs. SSDE
Schirmer	Mean ± SD (mm)	5.72 ± 6.25	6.68 ± 8.63	≥10	*P* = 0.018 (NNSDE vs. NSSDE)
FBUT	Mean ± SD (s)	2.32 ± 1.34	4.71 ± 2.79	≥10	*P* <0.001, SSDE vs. NSSDE
Cornea staining score	Mean ± SD	6.67 ± 3.32	2.32 ± 1.03	0	*P* <0.001, SSDE vs. NSSDE

### Next Generation Sequencing (NGS) Data

A total of 5,368,240 high-quality sequences were generated from the 98 samples of conjunctival sac swabs, with an average of 54,778 sequences per sample. High-quality sequences were clustered into 5,031 OTUs at 97% sequence identity. A modified OTU table was obtained consisting of 4,904 OTUs (ranging from 36 to 693 per sample), corresponding to 51 phyla, 142 classes, 315 orders, 523 families, and 1,028 genera.

The OTUs were compared with a Venn diagram (Supplementary Figure S1) and 811 common OTUs were identified. There were 60 (2,090/3,512), 22 (544/2,442), and 17% (306/1,816) OTUs that were unique in the NC group, NSSDE group, and SSDE group, respectively.

### Alpha and Beta Diversity Analysis

The goods coverage values were all ≥0.99, indicating that the 16S rRNA sequencing results from each library represented the majority of bacterial species present within test samples. The species accumulation curves flattened (Supplementary Figure S2), demonstrating that the sample size is adequate to represent the overall bacterial diversity in the target population.

**Figure 1:**
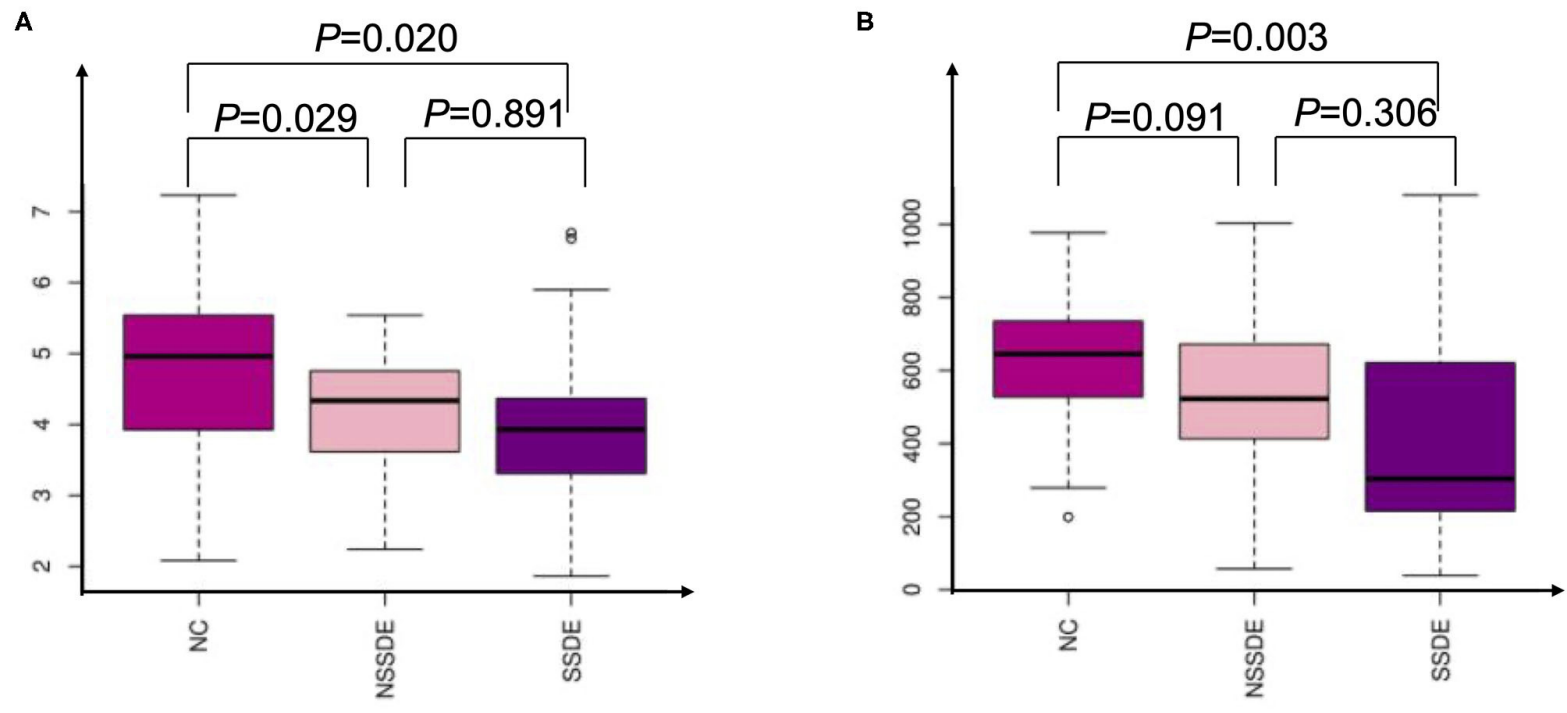
Alpha analysis by Shannon **(A)** and Chao1 **(B)** indexes demonstrating that patients with dry eyes have diminished ocular surface microbiome diversity compared with patients with healthy ocular surfaces.

The alpha diversity was analyzed by the Shannon and chao1 indexes. The Shannon index was significantly lower in the SSDE (*P* = 0.020) and NSSDE (*P* = 0.029) groups compared with the NC group but did not show a statistically significant difference between the NSSDE group and the SSDE group (*P* = 0.089) (Figure 1A). The chao1 index showed the same tendency as the Shannon index (NC vs. SSDE: *P* = 0.003, NC vs. NSSDE: *P* = 0.091, NSSDE vs. SSDE: *P* = *0.0*36) (Figure 1B).

**Figure 2:**
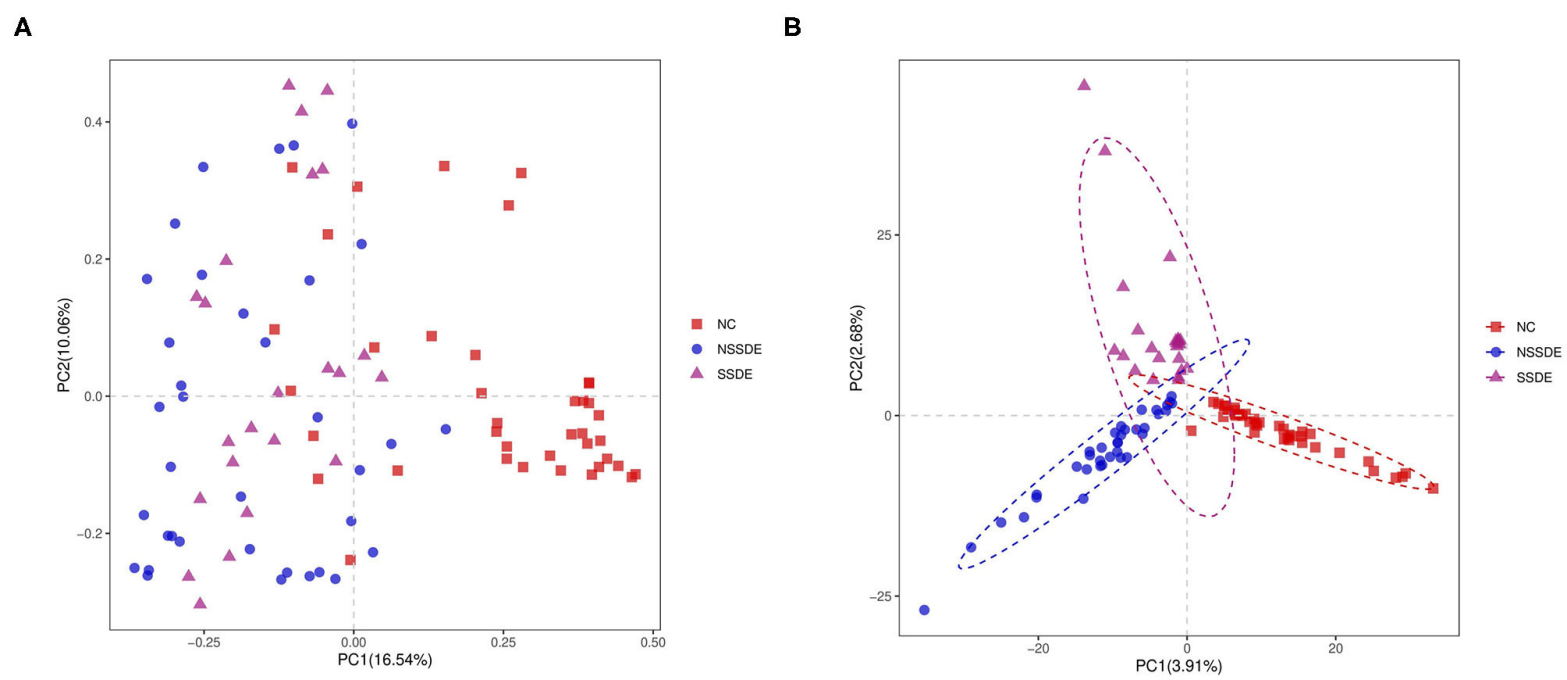
Beta diversity demonstrated by principal coordinates analysis (PCoA) **(A)** and Partial Least Squares Discrimination Analysis (PLS-DA) **(B)** analysis. Samples in the control group were more centralized and resembled each other in the bacterial composition, while samples in the non-Sjögren's syndrome-associated dry eyes (NSSDE) and Sjögren's syndrome-associated dry eyes (SSDE) groups were more acentric and disperse.

There was significant divergence in beta diversity between each group as demonstrated by PCoA (Figure 2A) and PLS-DA (Figure 2B). The samples in the control group were more centralized and resembled each other in the bacterial composition, while the samples in the NSSDE and SSDE groups were more acentric and disperse. This differentiation was further confirmed by ANOSIM analysis, which showed the difference between the groups was greater than the difference within the group (NC vs. SSDE: *P* = 0.001, NC vs. NSSDE: *P* = 0.001, NSSDE vs. SSDE: *P* = 0.005) (Table 2).

**Table 2:** Analysis of similarity (ANOSIM) for diversity between groups.

**Group**	**R statistic**	***P*-value**
NC-NSSDE	0.5485	0.001
NC-SSDE	0.6064	0.001
NSSDE-SSDE	0.1195	0.005
all	0.4428	0.001

*R value >0 means that differences between groups are greater than differences within groups*.

### Bacterial Relative Predominance

**Figure 3:**
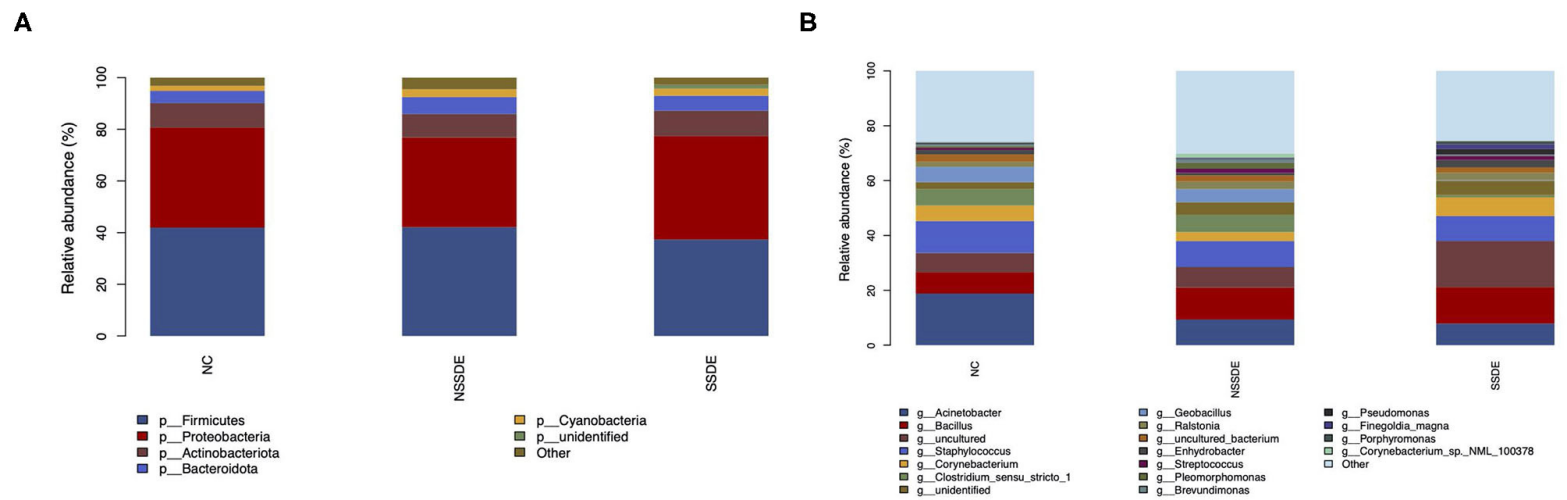
The top five abundant microbiomes at the phylum **(A)** and genus **(B)** level showed similar major components of bacteria in each group, but the relative abundance was different.

We summarized the relative abundances of the dominant bacterial community in each group. At the phylum level, 38 phyla were detected from the 23 eyes of the SSDE group, 36 phyla from the 36 eyes of the NSSDE group, and 48 phyla from the 39 eyes of the NC group. The top 5 abundant phyla were Firmicutes, Proteobacteria, Actinobacteriota, Bacteroidota, and Cyanobacteria in all three groups (Figure 3A). The relative percentage of each predominant phylum was demonstrated in Table 3. The Firmicutes/Bacteroidetes (F/B) ratio, which was correlated with inflammation in gut microbiome studies, was calculated. The F/B ratio was 6.42, 7.31, and 9.71 in the NC, NSSDE, and SSDE groups, respectively (*P* = 0.038, SSDE vs. NC, *P* = 0.991, NSSDE vs. NC, *P* = 0.048, SSDE vs. NSSDE).

**Table 3:** Percentage of the top five phyla in each group.

**Phylum**	**NC (%)**	**NSSDE (%)**	**SSDE (%)**	***P-*** **value**		
				**NC vs. NSSDE**	**NC vs. SSDE**	**NSSDE vs. SSDE**
Firmicutes	43.89	37.97	40.52	0.155	0.288	0.706
Proteobacteria	34.78	43.61	33.04	0.014	0.396	0.014
Actinobacteriota	6.60	8.24	15.89	0.020	0.281	0.005
Bacteroidota	6.84	5.19	4.17	0.110	0.027	0.250
Cyanobacteria	4.35	1.51	0.96	<0.001	0.005	0.680

**Figure 4:**
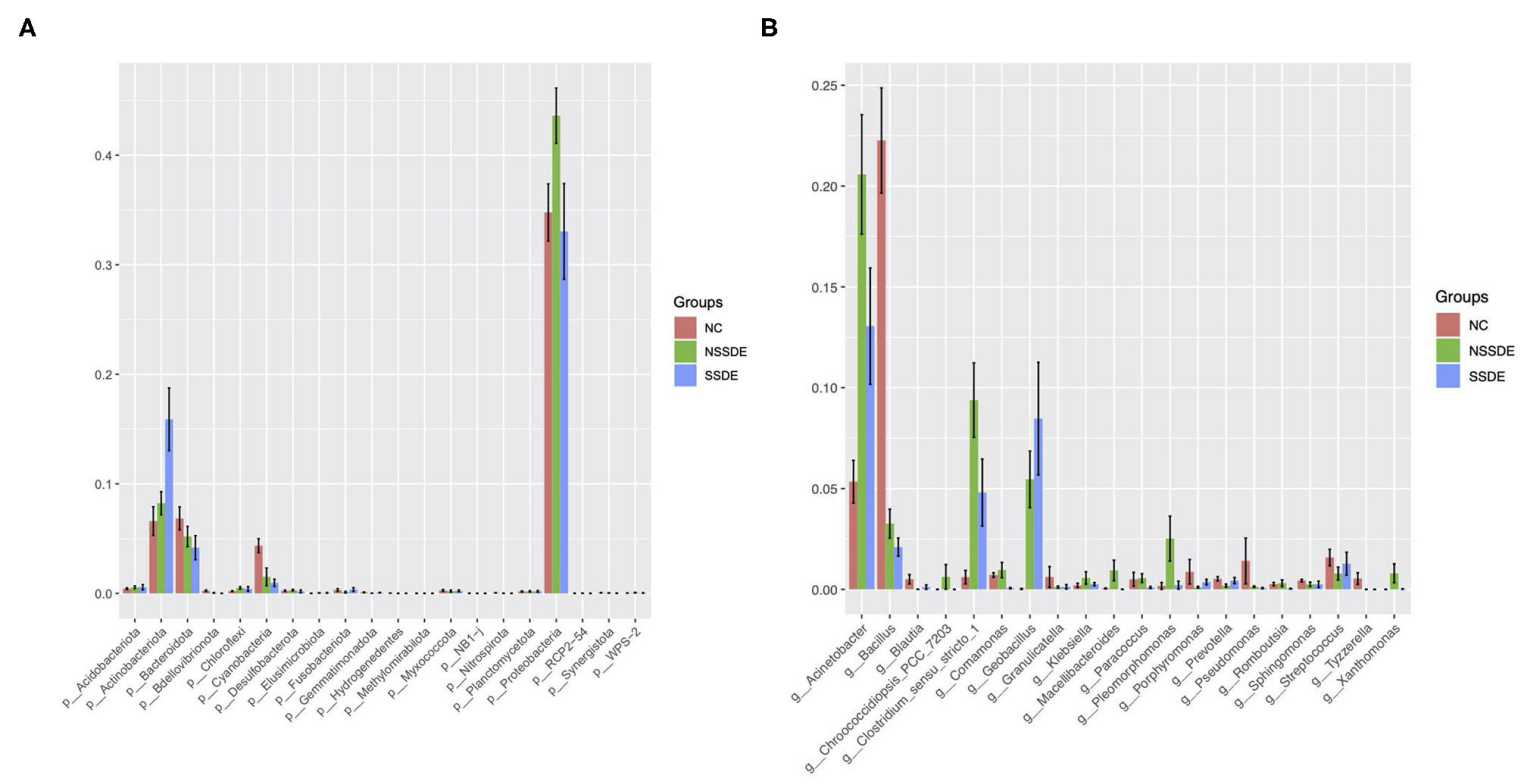
The top 20 significantly different microbiomes at the phylum **(A)** and genus **(B)** level.

Among the most abundant phyla, the relative abundance was also different between groups (Figure 4A). The relative abundance of Actinobacteriota was higher in the SSDE group (*P* = 0.005, SSDE vs. NSSDE, *P* < 0.001, SSDE vs. NC), followed by the NSSDE group (*P* = 0.020, NC vs. NSSDE) and NC group. Cyanobacteria were more abundant in the NC group (*P* <0.001, NC vs. NSSDE, *P* < 0.001, NV vs. SSDE), followed by the NSSDE group and SSDE group (*P* = 0.680). Bacteroidota was higher in abundance in the NC group than SSDE (*P* = 0.011), but there was no statistical difference between NC and NSSDE (*P* = 0.110) or NSSDE and SSDE (*P* = 0.250). Proteobacteria was more abundant in the NSSDE group than the SSDE group (*P* = 0.014) and NC group (*P* = 0.042), but its relative abundance was similar between the SSDE and NC group (*P* = 0.443).

At the genus level, 476 genera were detected in the SSDE group, 615 genera in the NSSDE group, and 928 genera in the NC group. The top five common abundant genera were *Acinetobacter, Staphylococcus, Bacillus, Corynebacterium*, and *Clostridium_sensu_stricto_1* (Figure 3B). And the relative percentage of each predominant genera was demonstrated in Table 4. *Bacillus* was significantly higher in the NC group compared with the dry eye groups (*P* < 0.001, NC vs. NSSDE, *P* < 0.001, NC vs. SSDE), whereas *Acinetobacter* was significantly higher in the dry eye groups than the NC group (*P* < 0.001, NC vs. NSSDE, *P* = 0.001, NC vs. SSDE). The relative abundance of *Acinetobacter* and *Bacillus* was similar between the SSDE and NSSDE groups (*P* = 0.096 for *Acinetobacter, P* = 0.727 for Bacillus). *Corynebacterium, Clostridium_sensu_stricto_1*, and *Geobacillus* were more abundant in the DED groups than the NC (*Corynebacterium*: *P* = 0.003, SSDE vs. NC, *P* < 0.001, NSSDE vs. NC; *Clostridium_sensu_stricto_1*: *P* = 0.039, SSDE vs. NC, *P* < 0.001, NSSDE vs. NC; *Geobacillus*: *P* < 0.001, SSDE vs. NC, *P* < 0.001, NSSDE vs. NC). The top 20 genera with the most significant differences were demonstrated in Figure 4B.

**Table 4:** Percentage of the top five genus in each group.

**Genus**	**NC (%)**	**NSSDE (%)**	**SSDE (%)**	***P*** **-value**		
				**NC vs. NSSDE**	**NC vs. SSDE**	**NSSDE vs. SSDE**
*Acinetobacter*	5.34	20.58	13.05	<0.001	0.001	0.096
*Staphylococcus*	7.14	11.43	13.55	0.316	0.543	0.859
*Bacillus*	22.26	3.26	2.09	<0.001	<0.001	0.727
*Corynebacterium*	3.82	4.15	8.62	0.076	0.003	0.050
*Clostridium_sensu_stricto_1*	0.61	9.38	4.80	<0.001	0.003	0.019

## Discussion

In recent years, the association of dysbiosis with inflammation and infection has become increasingly recognized (16). On the ocular surface, the antimicrobial activity of tears prevents infection while maintaining a commensal bacterial population (17). In this study, we sampled the conjunctival sac to explore the ocular surface microbiome composition and showed that dry eyes with and without SS have less microbiome diversity than eyes with healthy ocular surface conditions, and have characteristic microbiome composition.

In previous studies, the richness of the conjunctival sac microbiome in patients with dry eyes was inconsistent. While some studies revealed an increased number of bacteria (18, 19), most studies were in accordance with our study that patients with dry eyes have diminished ocular surface microbiome diversity (20–23). In studies regarding SS, bacterial diversity was diminished both for the oral mucosa (24) and the ocular surface (23). The exact mechanism is still unclear but might be an interaction between the mucosa and the commensal bacteria. The microbiome on the ocular surface degrades intracellular mucins, which in turn inhibit bacterial growth, contributing to homeostasis between the ocular microenvironment and commensal bacteria (17). Disruption of either the two ends of homeostasis might cause unbalanced growth of bacteria. In this study, while the major microbiome was stable, the diversity was severely diminished and an aberrant bacterial composition in dry eye patients was demonstrated by the observed clustering and separation demonstrated by the PCoA and PLS-DA analysis.

At the phylum level, Firmicutes, Proteobacteria, Actinobacteriota, Bacteroidota, and Cyanobacteria were the top five phyla in all three groups. These results were consistent with other studies that collect samples from the conjunctival sac (22, 25–27), indicating a relative stable predominant phylum composition of the ocular surface even in different disease status. However, there was a common issue that needs to be addressed in these studies, including ours, that there was no blank control to rule out environmental contamination. Cyanobacteria is photosynthetic and was not supposed to be found in the conjunctival sac which has no sun exposure. One possible explanation was that there might be Cyanobacteria on the ocular surface and dropped into the conjunctival sac. The 16srRNA method could also identify dead microbiomes as well. Thus, Cyanobacteria might also be detected. Supplementary experiments in further studies are needed to give a more pronounced answer.

The relative abundance at the phylum level was different in different groups. Actinobacteriota was more predominant in patients with dry eyes, while Cyanobacteria was less predominant, compared with healthy eyes. These differentiations were further magnified by the etiology of dry eyes. Patients with SSDE had more abundant Actinobacteriota and less Cyanobacteria compared with patients with NSSDE. We assumed that this might be associated with the severity of dry eye, as Schirmer's test, FBUT test, and cornea staining all showed a more severe degree of dry eye damage to the ocular surface. Proteobacteria was markedly higher in the NSSDE group compared with the SSDE group, and this was in accordance with the other study addressing the different microbiome compositions in DED with and without the autoimmune disease (23).

Although the relative abundance of Firmicutes and Bacteroides were similar in these three groups, we calculated the F/B ratio, inspired by the fact that a lower F/B ratio in the gut was associated with the inflammation status in the intestine such as in inflammatory bowel disease (28) and other systemic diseases such as systemic lupus erythematosus (29), while a higher F/B ratio in the gut was associated with obesity (28). Previous studies also showed that the F/B ratio in the gut was lower in patients with SSDE than in NC (30). However, in this study, we showed that the F/B ratio of the conjunctival sac was higher in the SSDE group compared with NC and NSSDE groups, in contrast with the gut F/B ratio. The F/B ratio change in both directions is a sign of dysbiosis (28). It is hypothesized that disrupted microbiome composition might activate distinct immunomodulatory and inflammatory pathways, which result in the instability of tear film and ocular damage. But we have to acknowledge that the gut has a large amount of microbial load while the ocular surface microbiome is paucibacterial. Thus, the effect of microbiome dysbiosis on the ocular surface might be less predominant. Further studies are needed to provide more evidence on the F/B ratio change and its relationship with SSDE and NSSDE.

At the gene level, patients with dry eyes had less genus variety. The most common five abundant genera were *Acinetobacter, Staphylococcus, Bacillus, Corynebacterium*, and *Clostridium_sensu_stricto_1* (Figure 3B), similar to the results of other studies concerning the ocular surface bacteria (22, 23, 25–27, 31). Compared with the NC group, patients with dry eyes had more abundant *Acinetobacter* but less *Bacillus*. *Acinetobacter* was commonly detected on the ocular surface of critically ill patients, who were always sedated and had unhealthy ocular surface function (32). However, previous studies showed that *Bacilli* are prominent in patients with dry eye disease, in contrast with the decreased Bacilli in DED groups in our study. Certain *Bacillus* species can synthesize and secrete lipopeptides, in particular, surfactins and mycosubtilins (33, 34), which might affect tear film stability. Further research needs to be done to explain the *Bacillus* variation in different studies and its role in dry eye disease.

*Corynebacterium* was significantly higher in abundance in the SSDE group than the NSSDE group, which was in line with the study that compares bacteria composition in patients with dry eyes with and without autoimmune diseases (23). This provided further evidence that *Corynebacterium* might be associated with autoimmunity, as mycolic acids and the cell wall architecture of *Corynebacterium* can affect macrophage function (35). However, there was also a study demonstrating that a decreased amount of *Corynebacterium* in patients with meibomian gland dysfunction (MGD) type of dry eye (36), supported by evidence that *Corynebacterium* elicited interleukin 17 response from γδ T cells in the ocular mucosa, driving neutrophil recruitment and antimicrobials release into the tears (37). These pieces of evidence were not contradictory, as the former one was a comparison between the etiology of dye eye disease, while the latter focuses on the inflammation status of MGD. *Clostridium_sensu_stricto_1* and *Geobacillus* were also higher in the dry eye groups in our study, but the association with functional microbiology and clinical significance need to be further addressed.

In previous studies regarding MGD, the overgrowth of *Staphylococcus* and *Sphingomonas* were detected (36), however, in this study, the amount of *Staphylococcus* was similar in the three groups; *Sphingomonas* was more abundant in the NC group. The different findings might be caused by different diagnostic criteria for DED, sampling methods (25), as well as different seasonal regions patients come from (38).

This study has several limitations. First, the F/B ratio at the phylum level was proposed for the first time in our study regarding the field of conjunctival sac microbiome. Although patients in the SSDE group have significantly higher F/B, the intrinsic association with SSDE and its opposite change in the gut of patients with SSDE need to be further investigated. Secondly, this was a single-center study that recruits patients from a certain area with a small sample size. The enrolled population was also homogeneous (mainly Han Chinese) to provide generalized conclusions. Further studies involving more diverse populations are expected. Thirdly, as SSDE more commonly affects mid-aged women, it was hard to have SSDE samples from male patients to match the sex and age balance. The influence of age and sex on microbiome composition was controversial (22, 36, 39). Further studies were expected to address this issue.

## Conclusions

In conclusion, we identified that the diversity of ocular surface microbiome in patients with NSSDE and SSDE was diminished compared with NC. The main microbiome at the phylum and genus level was similar between groups, but the relative abundance of the top five phyla and genera had variations. F/B ratio was significantly higher in the SSDE group compared with NC and NSSDE group.

## Data Availability Statement

The datasets presented in this study can be found in online repositories. The names of the repository/repositories and accession number(s) can be found below: https://www.ncbi.nlm.nih.gov/bioproject/, PRJNA802065.

## Ethics Statement

The studies involving human participants were reviewed and approved by the Institutional Review Board of Peking Union Medical College Hospital. The patients/participants provided their written informed consent to participate in this study.

## Author Contributions

HS and KX wrote the manuscript. HS and QL analyzed the data. ZC applied for the ethical approval of the study. QL designed this study and was responsible for sample collection and also critically reviewed, edited, and finalized the manuscript for submission. All authors contributed to the article and approved the submitted version.

## Funding

This study was supported by the National Natural Science Foundation of China (Number 81870685) and the Natural Science Foundation of Beijing Municipality (Number 7172173).

## Conflict of Interest

The authors declare that the research was conducted in the absence of any commercial or financial relationships that could be construed as a potential conflict of interest.

## Publisher's Note

All claims expressed in this article are solely those of the authors and do not necessarily represent those of their affiliated organizations, or those of the publisher, the editors and the reviewers. Any product that may be evaluated in this article, or claim that may be made by its manufacturer, is not guaranteed or endorsed by the publisher.

## Supplementary Material

Supplementary Figure S1 Venn diagram showing the number of operational taxonomic units (OTUs) that each group consisted of and its relationship with other groups.

Supplementary Figure S2 Species accumulation analysis showing the increase in OTUs detected with the addition of each sample.

## References

[1] Messmer EM. The pathophysiology, diagnosis, and treatment of dry eye disease. Deutsches Arzteblatt Int. (2015) 112:71–81. 10.3238/arztebl.2015.0071 25686388 PMC4335585

[2] Baudouin C Messmer EM Aragona P Geerling G Akova YA Benítez-del-Castillo J. Revisiting the vicious circle of dry eye disease: a focus on the pathophysiology of meibomian gland dysfunction. Br J Ophthalmol. (2016) 100:300–6. 10.1136/bjophthalmol-2015-307415 26781133 PMC4789719

[3] O'Neil EC Henderson M Massaro-Giordano M Bunya VY. Advances in dry eye disease treatment. Curr Opin Ophthalmol. (2019) 30:166–78. 10.1097/ICU.0000000000000569 30883442 PMC6986373

[4] Buckley RJ. Assessment and management of dry eye disease. Eye. (2018) 32:200–3. 10.1038/eye.2017.289 29303149 PMC5811740

[5] Both T Dalm VA van Hagen PM van Daele PL. Reviewing primary Sjögren's syndrome: beyond the dryness - from pathophysiology to diagnosis and treatment. Int J Med Sci. (2017) 14:191–200. 10.7150/ijms.17718 28367079 PMC5370281

[6] Kuklinski E Asbell PA. Sjogren's syndrome from the perspective of ophthalmology. Clin Immunol. (2017) 182:55–61. 10.1016/j.clim.2017.04.017 28476437

[7] Bjordal O Norheim KB Rødahl E Jonsson R Omdal R. Primary Sjögren's syndrome and the eye. Surv Ophthalmol. (2020) 65:119–32. 10.1016/j.survophthal.2019.10.004 31634487

[8] Vehof J Utheim TP Bootsma H Hammond CJ. Advances, limitations and future perspectives in the diagnosis and management of dry eye in Sjögren's syndrome. Clin Exp Rheumatol. (2020) 38(Suppl. 126):301–9. 33025899

[9] Harmsen HJ de Goffau MC. The Human Gut Microbiota. Adv Exp Med Biol. (2016) 902:95–108. 10.1007/978-3-319-31248-4_7 27161353

[10] Dinan TG Cryan JF. Brain-gut-microbiota axis and mental health. Psychosom Med. (2017) 79:920–6. 10.1097/PSY.0000000000000519 28806201

[11] Aristoteli LP Bojarski B Willcox MD. Isolation of conjunctival mucin and differential interaction with Pseudomonas aeruginosa strains of varied pathogenic potential. Exp Eye Res. (2003) 77:699–710. 10.1016/j.exer.2003.08.007 14609558

[12] Mantelli F Argueso P. Functions of ocular surface mucins in health and disease. Curr Opin Allergy Clin Immunol. (2008) 8:477–83. 10.1097/ACI.0b013e32830e6b04 18769205 PMC2666617

[13] Liu ZG Sun XG Zhang MC Xu JJ Hong J Deng YP. Asia dry eye association, professional committee of ophthalmology of cross-strait medical and health exchange association, Chinese branch, ophthalmology and dryness group, ophthalmology and dryness group, ophthalmology branch of Chinese medical doctor association (in Chinese with english abstract). J Ophthalmol. (2020) 56:741–7. 10.3760/cma.j.cn112142-20200714-00477

[14] Lemp MA. Report of the National Eye Institute/Industry workshop on Clinical Trials in Dry Eyes. CLAO J. (1995) 21:221–32. 8565190

[15] Shiboski CH Shiboski SC Seror R Criswell LA Labetoulle M Lietman TM. 2016 American College of Rheumatology/European League Against Rheumatism classification criteria for primary Sjögren's syndrome: a consensus and data-driven methodology involving three international patient cohorts. Ann Rheum Dis. (2017) 76:9–16. 10.1136/annrheumdis-2016-210571 27789466

[16] Levy M Kolodziejczyk AA Thaiss CA Elinav E. Dysbiosis and the immune system. Nat Rev Immunol. (2017) 17:219–32. 10.1038/nri.2017.7 28260787

[17] Berry M Harris A Lumb R Powell K. Commensal ocular bacteria degrade mucins. Br J Ophthalmol. (2002) 86:1412–6. 10.1136/bjo.86.12.1412 12446377 PMC1771402

[18] Graham JE Moore JE Jiru X Moore JE Goodall EA Dooley JS. Ocular pathogen or commensal: a PCR-based study of surface bacterial flora in normal and dry eyes. Invest Ophthalmol Vis Sci. (2007) 48:5616–23. 10.1167/iovs.07-0588 18055811

[19] Jiang X Deng A Yang J Bai H Yang Z Wu J. Pathogens in the Meibomian gland and conjunctival sac: microbiome of normal subjects and patients with Meibomian gland dysfunction. Infect Drug Resist. (2018) 11:1729–40. 10.2147/IDR.S162135 30349330 PMC6188152

[20] Li Z Gong Y Chen S Li S Zhang Y Zhong H. Comparative portrayal of ocular surface microbe with and without dry eye. J Microbiol. (2019) 57:1025–32. 10.1007/s12275-019-9127-2 31463790

[21] Zhao F Zhang D Ge C Zhang L Reinach PS Tian X. metagenomic profiling of ocular surface microbiome changes in meibomian gland dysfunction. Invest Ophthalmol Vis Sci. (2020) 61:22. 10.1167/iovs.61.8.22 32673387 PMC7425691

[22] Andersson J Vogt JK Dalgaard MD Pedersen O Holmgaard K Heegaard S. Ocular surface microbiota in patients with aqueous tear-deficient dry eye. Ocul Surf. (2021) 19:210–7. 10.1016/j.jtos.2020.09.003 32931939

[23] Qi Y Wan Y Li T Zhang M Song Y Hu Y. Comparison of the ocular microbiomes of dry eye patients with and without autoimmune disease. Front Cell Infect Microbiol. (2021) 11:716867. 10.3389/fcimb.2021.716867 34631599 PMC8493086

[24] Zhou Z Ling G Ding N Xun Z Zhu C Hua H. Molecular analysis of oral microflora in patients with primary Sjogren's syndrome by using high-throughput sequencing. PeerJ. (2018) 6:e5649. 10.7717/peerj.5649 30280027 PMC6166617

[25] Dong Q Brulc JM Iovieno A Bates B Garoutte A Miller D. Diversity of bacteria at healthy human conjunctiva. Invest Ophthalmol Vis Sci. (2011) 52:5408–13. 10.1167/iovs.10-6939 21571682 PMC3176057

[26] Lee SH Oh DH Jung JY Kim JC Jeon CO. Comparative ocular microbial communities in humans with and without blepharitis. Invest Ophthalmol Vis Sci. (2012) 53:5585–93. 10.1167/iovs.12-9922 22836761

[27] Zhou Y Holland MJ Makalo P Joof H Roberts CH Mabey DC. The conjunctival microbiome in health and trachomatous disease: a case control study. Genome Med. (2014) 6:99. 10.1186/s13073-014-0099-x 25484919 PMC4256740

[28] Stojanov S Berlec A Strukelj B. The influence of probiotics on the firmicutes/bacteroidetes ratio in the treatment of obesity and inflammatory bowel disease. Microorganisms. (2020) 8. 10.3390/microorganisms8111715 33139627 PMC7692443

[29] De Luca F Shoenfeld Y. The microbiome in autoimmune diseases. Clin Exp Immunol. (2019) 195:74–85. 10.1111/cei.13158 29920643 PMC6300652

[30] Mendez R Watane A Farhangi M Cavuoto KM Leith T Budree S. Gut microbial dysbiosis in individuals with Sjogren's syndrome. Microb Cell Fact. (2020) 19:90. 10.1186/s12934-020-01348-7 32293464 PMC7158097

[31] Zilliox MJ Gange WS Kuffel G Mores CR Joyce C de Bustros P. Assessing the ocular surface microbiome in severe ocular surface diseases. Ocul Surf. (2020) 18:706–12. 10.1016/j.jtos.2020.07.007 32717380 PMC7905829

[32] Mela EK Drimtzias EG Christofidou MK Filos KS Anastassiou ED Gartaganis SP. Ocular surface bacterial colonisation in sedated intensive care unit patients. Anaesth Intensive Care. (2010) 38:190–3. 10.1177/0310057X1003800129 20191796

[33] Favaro G Bogialli S Di Gangi IM Nigris S Baldan E Squartini A. Characterization of lipopeptides produced by *Bacillus licheniformis* using liquid chromatography with accurate tandem mass spectrometry. Rapid Commun Mass Spectrom. (2016) 30:2237–52. 10.1002/rcm.7705 27487987

[34] Nigris S Baldan E Tondello A Zanella F Vitulo N Favaro G. Biocontrol traits of *Bacillus licheniformis* GL174, a culturable endophyte of *Vitis vinifera* cv. Glera BMC Microbiol. (2018) 18:133. 10.1186/s12866-018-1306-5 30326838 PMC6192205

[35] Burkovski A. The role of corynomycolic acids in Corynebacterium-host interaction. Antonie Van Leeuwenhoek. (2018) 111:717–25. 10.1007/s10482-018-1036-6 29435693

[36] Dong X Wang Y Wang W Lin P Huang Y. Composition and diversity of bacterial community on the ocular surface of patients with meibomian gland dysfunction. Invest Ophthalmol Vis Sci. (2019) 60:4774–83. 10.1167/iovs.19-27719 31738825

[37] St Leger AJ Desai JV Drummond RA Kugadas A Almaghrabi F Silver P. An Ocular commensal protects against corneal infection by driving an interleukin-17 response from mucosal gammadelta T cells. Immunity. (2017) 47:148–58.e5. 10.1016/j.immuni.2017.06.014 28709803 PMC5553552

[38] Ozkan J Nielsen S Diez-Vives C Coroneo M Thomas T Willcox M. Temporal stability and composition of the ocular surface microbiome. Sci Rep. (2017) 7:9880. 10.1038/s41598-017-10494-9 28852195 PMC5575025

[39] Wen X Miao L Deng Y Bible PW Hu X Zou Y. The influence of age and sex on ocular surface microbiota in healthy adults. Invest Ophthalmol Vis Sci. (2017) 58:6030–7. 10.1167/iovs.17-22957 29196767

