# Supplementary figures and images for: Analysis of Conjunctival Sac Microbiome in Dry Eye Patients With and Without Sjögren's Syndrome
Source: Front Med (Lausanne). 2022 Mar 8;9:841112. doi: 10.3389/fmed.2022.841112 (PMC8957797; doi:10.3389/fmed.2022.841112)

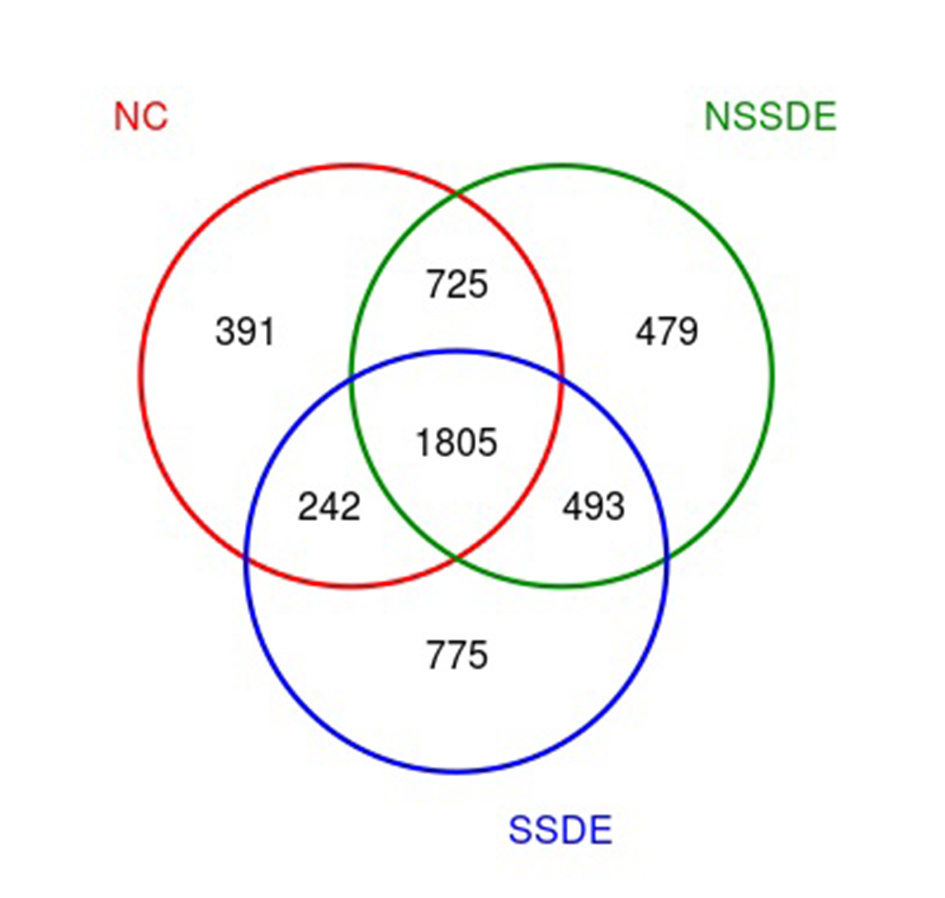

Supplement: Supplementary Figure S1 — Venn diagram showing the number of operational taxonomic units (OTUs) that each group consisted of and its relationship with other groups. [file Image_1.JPEG]

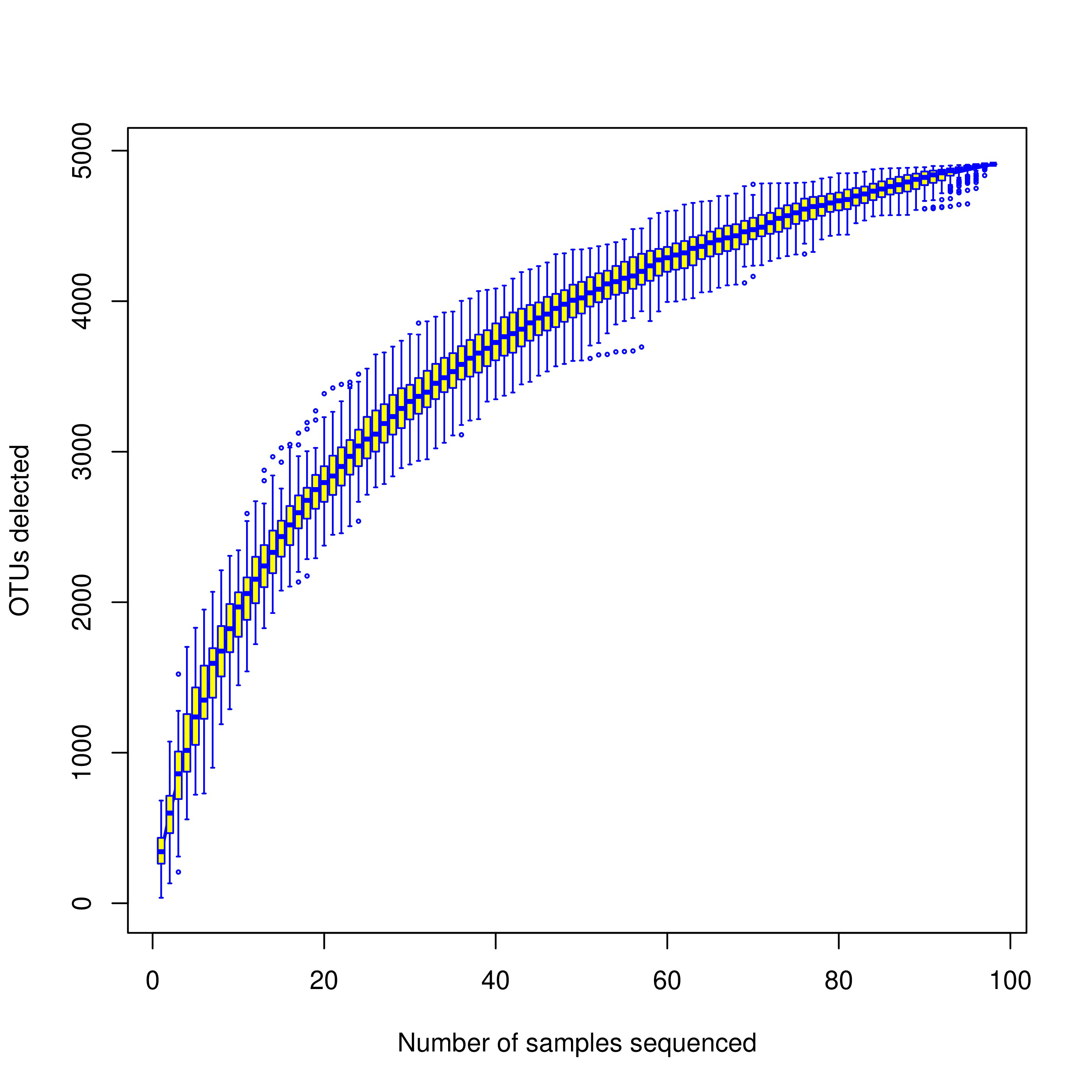

Supplement: Supplementary Figure S2 — Species accumulation analysis showing the increase in OTUs detected with the addition of each sample. [file Image_2.JPEG]
